# Hematopoietic islands mimicking osteoblastic metastases within the axial skeleton

**DOI:** 10.1186/s12891-022-05402-w

**Published:** 2022-05-12

**Authors:** Sophia Samira Goller, Bernd Erber, Nicola Fink, Hans Roland Dürr, Thomas Knösel, Jens Ricke, Andrea Baur-Melnyk

**Affiliations:** 1 Department of Radiology, University Hospital, LMU Munich, 81377 Munich, Germany; 2 Department of Orthopaedics and Trauma Surgery, Musculoskeletal University Center Munich (MUM), University Hospital, LMU Munich, 81377 Munich, Germany; 3 Institute of Pathology, University Hospital, LMU Munich, 81377 Munich, Germany

**Keywords:** Hyperplasia of the hematopoietic bone marrow, Hematopoietic islands, Axial skeleton, Osteoblastic metastases, MRI, CT

## Abstract

**Background:**

Hyperplasia of the hematopoietic bone marrow in the appendicular skeleton is common. In contrast, focal hematopoietic islands within the axial skeleton are a rare entity and can confuse with osteoblastic metastases. This study aimed to characterize typical MRI and CT findings of hematopoietic islands in distinction from osteoblastic metastases to help both radiologists and clinicians, on the one hand, not to overdiagnose this entity and, on the other hand, to decide on a reasonable work-up.

**Methods:**

We retrospectively analyzed the imaging findings of 14 hematopoietic islands of the axial skeleton in ten patients (nine females, median age = 65.5 years [range, 49–74]) who received both MRI and CT at initial diagnosis between 2006 and 2020. CT-guided biopsy was performed in five cases to confirm the diagnosis, while the other five patients received long-term MRI follow-up (median follow-up = 28 months [range, 6–96 months]). Diffusion-weighted imaging was available in three, chemical shift imaging respectively ^18^F- fluorodeoxyglucose PET/CT in two, and Technetium 99 m skeletal scintigraphy in one of the patients.

**Results:**

All lesions were small (mean size = 1.72 cm^2^) and showed moderate hypointense signals on T1- and T2-weighted MRI sequences. They appeared isointense to slightly hyperintense on STIR images and slightly enhanced after gadolinium administration. To differentiate this entity from osteoblastic metastases, CT provides important additional information, as hematopoietic islands do not show sclerosis.

**Conclusions:**

Hematopoietic islands within the axial skeleton can occur and mimic osteoblastic metastases. However, the combination of MRI and CT allows for making the correct diagnosis in most cases.

## Background

While bone marrow in the fetus is entirely hematopoietic, conversion to fatty marrow begins in the distal extremities soon after birth and is confined to the axial skeleton, proximal humeri, femoral neck, and intertrochanteric regions by young adulthood. The hematopoietic marrow itself is composed of cellular components and varying amounts of fat, whereby the fat content increases with age [1]. Mild forms of hyperplasia of the red bone marrow are associated with heavy smoking, long-distance running, and obesity [2–4]. More severe and diffuse forms can be related to chronic anemias (particularly hematolytic types) and benign and malignant infiltrative bone marrow disorders, such as Gaucher disease, myelofibrosis, myeloma, and lymphoma, leukemia, and metastatic disease [2, 5, 6]. Besides, hematopoietic marrow hyperplasia can be encountered after administering granulocyte-colony-stimulating factors [7, 8].

Since peripheral manifestations of hematopoietic bone marrow hyperplasia are significantly more common than those of the axial skeleton, these foci are often accidentally seen on routine MRI of the lower extremities, in particular around the knee joint, as previously described in a case series of Deutsch et al. [2]. However, focal hyperplasia of the red bone marrow has been found within the axial skeleton, described in a case report by Bordalo-Rodrigues et al. in 2002 [9]. Other examples given, two previous case reports described the detection of focal hematopoietic hyperplasia in the ribs [10, 11].

Against this background, diagnostic difficulties may arise in distinguishing focal hematopoietic hyperplasia of the axial skeleton (“hematopoietic islands”) from osteoblastic metastases, particularly when underlying malignancy is known.

Therefore, the purpose of our study was to evaluate and characterize typical MRI and CT findings of hematopoietic islands of the axial skeleton to help both radiologists and clinicians, on the one hand, not to overdiagnose this entity, on the other hand, to decide on a reasonable work-up. Newer imaging techniques, such as diffusion-weighted imaging (DWI), chemical shift imaging (CSI), and hybrid imaging techniques, were evaluated in a subgroup of our patients.

## Methods

### Patient population

This retrospective single-center analysis included ten patients with 14 hematopoietic islands of the axial skeleton who underwent both MRI and CT at the initial diagnosis between January 2006 and January 2020. Patients’ characteristics are summarized in Table 1. In detail, patients were retrospectively identified via a full-text query within the local radiology information system using the search term “hematopoietic island.” Subsequently, the resulting reports were further filtered concerning the availability of initial MRI and CT examinations and the presence of sufficiently long follow-up periods (at least six months) and/or histologically confirmed lesions. None of the tumor patients included had apparent osseous metastases. In two patients, long-term follow-up of 28 respectively 50 months was available despite the histopathological findings derived from CT-guided biopsy.

**Table 1:** Patients’ characteristics

Patient	**Age**	**Sex**	**Lesion localisation [n]**	**Lesion size**^a^	**Previous malignancy**	**Primary CT-guided biopsy**	**MRI follow-up [months]**
**1**	66	f	Thoracic spine [3] Lumbar spine [1]	0,8 × 0,6 1,0 × 1,0 1,6 × 1,2 1,6 × 1,6	Breast cancer	yes	28^b^
**2**	67	f	Os sacrum [1]	1,3 × 1,1	Gastric SRCC	no	96
**3**	64	f	Os sacrum [1]	1,1 × 1,2	Breast cancer	yes	
**4**	65	f	Os sacrum [1]	1,6 × 1,7	no	yes	
**5**	53	f	Rib [1]	1,8 × 0,8	no	yes	50 ^b^
**6**	50	f	Lumbar spine [1]	1,5 × 1,3	Breast cancer (IDC)	no	29
**7**	73	f	Lumbar spine [1]	1,0 × 0,9	no	no	20
**8**	72	f	Thoracic spine [1]	1,3 × 1,2	no	no	6
**9**	49	m	Lumbar spine [1] Os ilium [1]	1,0 × 1,0 1,2 × 1,3	no	yes	
**10**	74	f	Lumbar spine [1]	2,1 × 1,8	no	no	11
**Total [n]** 10	**Mean [years]** 63.3 (49–74) **Median [years]** 65.5	**Male/ female ratio [n]** 1/9	**Total [n]** 14	**Mean [cm**^**2**^**]** 1.72 (0.48–3.78) **Median [cm**^**2**^**]** 1.46	**Total [n]** 4	**Total [n]** 5	**Mean [months]** 34.2 (6–96) **Median [months]** 28

*f* Female, *m* Male, *SRCC* Signet ring cell carcinoma, *IDC* Invasive ductal carcinoma

^a^lesion size [cm x cm, axial T2- weighted images]

^b^additional MRI follow-up in patients who underwent CT-guided biopsy

### Imaging techniques

MR imaging was performed using a 1.5 Tesla unit (Avanto, Siemens Healthineers, Erlangen, Germany). MRI spine protocols routinely comprised the following pulse sequences: Sagittal T1-weighted turbo spin-echo (TSE) MR imaging (repetition time msec/ echo time msec: 400–700/ 8–16), sagittal T2-weighted TSE MR imaging (repetition time msec/ echo time msec: 5000–7000/80–120), sagittal short tau inversion recovery (STIR) TSE MR imaging (repetition time msec/ echo time msec/ inversion time msec: 2000–4000/20–40/ 150), and contrast-enhanced T1-weighted TSE MR imaging (repetition time msec/ echo time msec: 500–600/ 9–13). The slice thickness for standard morphological sequences varied between 3 and 4 mm. The field of view and matrix varied as being adapted to the individual distribution of the lesions. In three patients (patients 1, 8, and 10; Table 1), diffusion-weighted imaging using reversed fast imaging with steady-state free precession sequence (SSFP) was done (repetition time msec/ echo time msec: 25/ 7.17; slice thickness mm: 7). In two patients (patients 1 and 10; Table 1), sagittal in-phase (100–165/4.2; flip angle, 30°) and out-of-phase (100–165/2.1; flip angle, 30°) fast multiplanar spoiled gradient-echo chemical shift imaging was performed. All patients underwent high-resolution CT at least once at the initial diagnosis. In addition, two of the patients (patients 1 and 2; Table 1) received ^18^F- fluorodeoxyglucose (FDG) PET/CT, and one (patient 1; Table 1) Technetium (Tc) 99 m skeletal scintigraphy.

The location and size (maximum diameter on axial T2-weighted images), as well as the signal intensities on all morphological MRI sequences, were evaluated, as well as bone texture changes (osteosclerosis/-lysis) on CT respectively FDG-uptake on PET/CT in two cases, and radiotracer accumulation on skeletal scintigraphy in one case. In addition, signal behavior on DWI and CSI was analyzed according to the known methods [12]. Using SSFP sequences, the signal intensity of each lesion was assessed qualitatively in relation to the adjacent bone marrow as either hypo-, iso- or hyperintense. According to Zajick et al., decreases in signal intensity greater than 20% on out-of-phase images compared with in-phase images have been used as a cut-off threshold for normalcy to allow distinction between benign (> 20% signal decrease on out-of-phase images) and malignant (< 20% signal decrease on out-of-phase images) marrow changes on CSI [13].

## Results

A total of 14 lesions in ten patients were analyzed. One patient showed four lesions, one patient showed two lesions respectively, while all other patients presented with one single lesion. The lesions were located at the thoracic spine (*n* = 4), lumbar spine (*n* = 5), Os sacrum (*n* = 3), Os ilium (*n* = 1), and the rib (*n* = 1). Lesion size ranged from 0.48–3.78 cm^2^ (mean: 1.72 cm^2^). All lesions had fairly sharp margins and were well demarcated from adjacent bone marrow. Detailed information on lesions’ localization and size is provided in Table 1.

### MRI

**Fig. 1:**
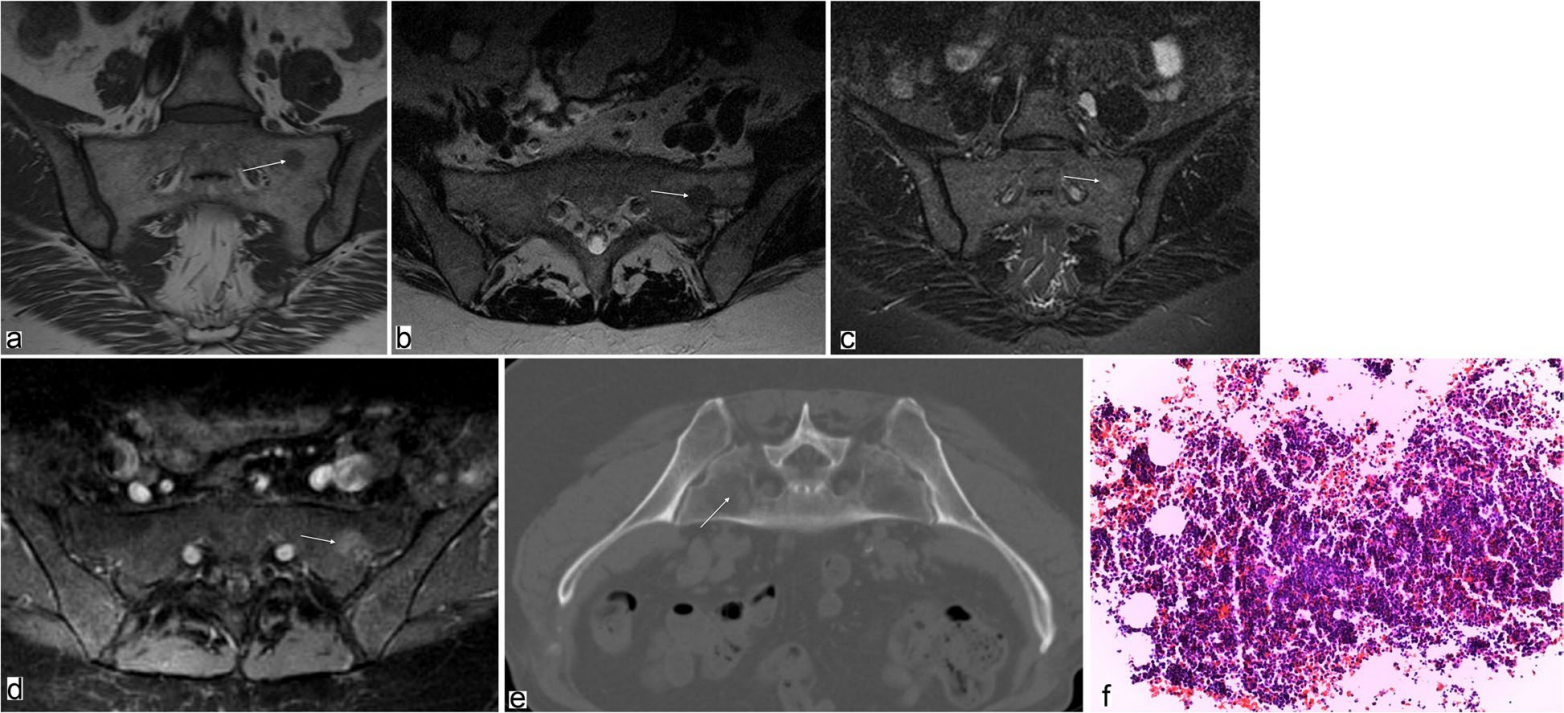
64-year-old woman with a history of breast cancer and focal hyperplasia of hematopoietic marrow in the massa lateralis of the sacrum. Arrows point to the lesion; H&E, hematoxylin and eosin. **a** Coronal T1-weighted TSE MR image of the sacral spine shows a focal area of moderately decreased signal intensity in the left massa lateralis. **b** Axial T2-weighted TSE image with a focal signal drop in the left massa lateralis (corresponding to a). **c** Slight hyperintense signal of the lesion is shown on the coronal STIR image. **d** On the axial contrast-enhanced fat-saturated T1-weighted image, the lesion shows slight enhancement. **e** High-resolution CT scan without any bone structure abnormalities (note: prone position before CT-guided biopsy). **f** Photograph of biopsy specimen (H&E, × 80) of the lesion shows hypercellular bone marrow with a reduced number of adipocytes. No neoplastic cells were found

**Fig. 2:**
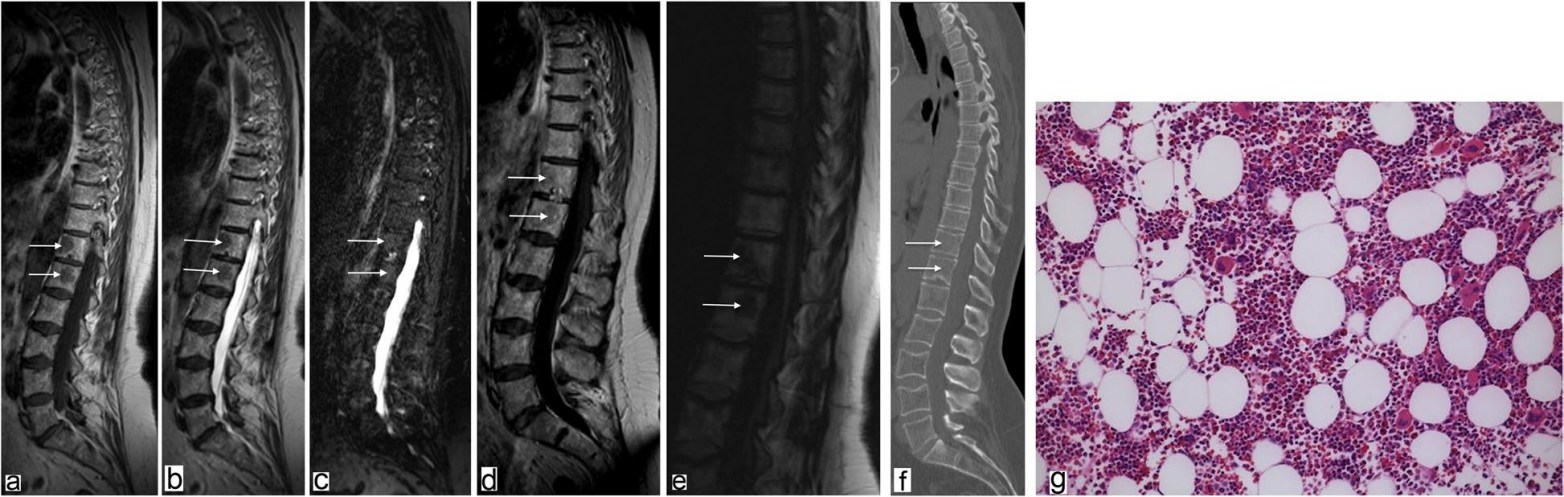
66-year-old woman with a history of breast cancer and presence of focal hyperplasia of hematopoietic marrow in the thoracic (Th10, Th11, Th12) and lumbar spine (L1). Images illustrate the Th12 and the L1 lesion (arrows); SSFP, steady-state free precession; H&E, hematoxylin and eosin. **a** Sagittal T1-weighted TSE MR image of the thoracic and lumbar spine shows focal areas of moderately decreased signal intensity in the vertebral bodies of Th12 and L1. **b** Sagittal T2-weighted TSE image with a signal drop in Th12 and L1 (corresponding to a). **c** Sagittal STIR image with slightly hyperintense signal in Th12 and L1 lesions. **d** Lesions show slight focal enhancement after gadolinium administration on a sagittal T1-weighted image. **e** Sagittal SSFP image with a hypointense signal of the Th12 and L1 lesion. **f** High-resolution CT scan without bone structure abnormalities in Th12 and L1. **g** Photograph of biopsy specimen (H&E, × 80) of the Th12 lesion shows hypercellular bone marrow with a reduced portion of adipocytes

Without exception, all lesions presented with moderate hypointense signals on T1- and T2-weighted images (*n* = 14) (Figs. 1a, b and 2a, b) while being isointense (*n* = 5) to slightly hyperintense on STIR-sequences (*n* = 9) (Figs. 1c and 2c). After gadolinium administration, all lesions (*n* = 13) showed mild enhancement (Figs. 1d and 2d). In *n* = 3 patients DWI with SSFP sequences was performed, where all lesions presented hypointensity compared to adjacent normal bone marrow (Fig. 2e). On CSI (*n* = 2), lesions showed a signal drop of greater than 20% on out-of-phase images compared with in-phase images, indicating fat within the lesions.

### CT

None of the hematopoietic islands showed abnormalities of the bone structure in terms of sclerotic or lytic changes on CT (Figs. 1e and 2f).

In summary, typical MRI and CT findings of hematopoietic islands in contrast to osteoblastic metastases are presented in Table 2.

**Table 2:** Characteristic imaging findings of hematopoietic islands and osteoblastic metastases

Imaging	Hematopoietic islands	Osteoblastic metastases
T1w	Slightly hypointense	Markedly hypointense
T2w	Slightly hypointense	Markedly hypointense
STIR	Iso- slightly hyperintense	Iso- hypointense
ce T1w	Slight enhancement	No/slight enhancement
CT	No sclerotic or lytic bone changes	Sclerosis

*w* Weighted, *STIR* short tau inversion recovery, *ce* Contrast-enhanced (gadolinium)

### PET-CT

In *n* = 2 patients, who received complementary ^18^F-FDG PET/CT, FDG- uptake was not increased (cut-off value SUV_max_ > 3 considered pathologic).

### Skeletal scintigraphy

Tc-99 m skeletal scintigraphy was unremarkable in *n* = 1 patient.

## Discussion

We presented characteristic MRI and CT findings of hematopoietic islands of the axial skeleton in a case series of ten patients. The awareness of this rare entity is essential to not misdiagnose these lesions as osteoblastic metastases, which typically show similar signal intensities on MRI. Diagnostic difficulties particularly may arise when underlying malignancy is known.

### MRI

Normal bone marrow shows intermediate signal intensity on T1-weighted spin-echo images since it contains about 50% fat and 50% water in adults [14]. In our patient cohort, all hematopoietic islands presented moderate hypointense signals on unenhanced T1-weighted TSE images compared to surrounding bone marrow. This is explained by the fact that a significant amount of fat is still preserved in hematopoietic islands. Our finding is consistent with previously reported cases of focal hematopoietic islands [2, 9–11]. All lesions described in the literature also presented mild hypointense signals on T1-weighted images, which helps differentiate these lesions from osteoblastic metastases, which usually show substantial T1 signal drop isointense or even hypointense compared to adjacent muscle or disk [15, 16].

Unenhanced T1-weighted sequences are essential in differentiating benign lesions with fat content (like benign bone marrow lesions or edema) from metastases, which generally show a significant reduction in fat component due to cellular replacement with marrow infiltration [17]. In a previous study, Carroll et al. analyzed T1-weighted images of 74 patients with both benign and malignant bone marrow signal alterations on MRI (51 biopsy-proven, 23 clinical follow-ups) and compared relative signal intensity of bone marrow to adjacent skeletal muscle and/or nondegenerated intervertebral disk to establish standards on MRI differentiating infiltrative marrow pathology from hematopoietic marrow. It was summarized that marrow lesions that are relatively isointense or hypointense to muscle and/or disk on T1-weighted spin-echo images should not be considered normal hematopoietic marrow [15].

In this context, Schweitzer et al. previously reported the “bull´s eye sign” as a specific indicator of normal hematopoietic marrow and the “halo sign” as a strong indicator of metastatic disease in 47 patients with osseous lesions of the pelvis evaluating T1- and T2-weighted sequences [18]. The “bull´s eye sign” which describes a central high T1 signal in an osseous lesion, could not be found in our cases of hematopoietic islands. Thus, T1-weighted images are essential for the differentiation of sclerotic osteoblastic metastases with a strong hypointense signal equal to adjacent disc or muscle and focal hematopoietic islands with only moderate signal drop.

Normal bone marrow shows intermediate signal intensity on T2-weighted images. All hematopoietic islands showed hypointense signals in our cohort on T2-weighted TSE images, similar to osteoblastic metastases. In contrast, osteolytic metastases show high signal intensity on T2-weighted images [14, 19, 20]. All previous reported hematopoietic islands showed hypointense T2-weighted signals, which is in concordance with our findings [2, 9–11].

STIR sequences provide high tissue contrast by suppressing fat signals. Thus, all pathologic processes, such as metastases, edema, and inflammation, show strong hyperintense signals. Normal bone marrow usually shows low signal intensity on STIR imaging [14, 20]. Five out of 14 lesions in our cohort showed isointense signals on STIR sequences compared to adjacent bone marrow, while nine showed slightly hyperintense signals. In contrast, osteolytic metastases typically show strong hyperintense signals on STIR sequences, while osteoblastic metastases show similar signal behavior to hematopoietic islands due to a lack of water protons [14]. Thus, T2-weighted and STIR sequences do not help differentiate focal hematopoietic islands from osteoblastic metastases.

After gadolinium administration, normal bone marrow enhances to a certain extent due to bone vascularity [21]. Therefore, spinal marrow contrast enhancement depends on age and fat content and is significantly higher in hematopoietic marrow, characterized by a high percentage of fenestrated vessels and a low amount of poorly vascularized fat [20, 22]. Interestingly, younger individuals may show a rate of signal intensity increase, which is within the range typical of diffuse malignant marrow infiltration [21]. The bone marrow enhancement decreases markedly with increasing age and fatty conversion, although significantly varying among individuals [22]. This may be explained by interindividual differences in hematopoietic and fat distribution and might be associated with arteriosclerotic changes altering marrow perfusion in the elderly [23]. This makes it difficult to distinguish normal from pathological enhancement patterns and has the consequence that contrast subtraction techniques have a limited significance. Typically, osteoblastic metastases show no or only slight contrast enhancement, while osteolytic lesions strongly enhance [14, 19, 20]. After contrast administration, all hematopoietic islands showed minor enhancement in our cohort, which was more conspicuous in fat-saturated images. Thus, gadolinium cannot differentiate between osteoblastic metastases and focal hematopoietic islands but helps differentiate hematopoietic islands from osteolytic or mixed type metastases, which usually show substantial enhancement. There are no previous studies describing the signal behavior of hematopoietic islands after gadolinium administration. However, contrast administration is highly recommended in uncertain bony lesions [24].

### Diffusion-weighted imaging

DWI is based on quantifying the motion of water molecules within tissue [25]. Three patients in our cohort received DWI with obtaining SSFP sequences with relatively short acquisition time and insensitivity to patient movement. All examined hematopoietic islands presented as hypointense lesions when qualitatively compared to adjacent bone marrow. In previous studies, SSFP imaging was able to differentiate between malignant, depicted as hyperintense, and benign vertebral fractures depicted as iso- or hypointense compared to normal bone marrow [25, 26]. However, osteoblastic metastases may also present hypointensity on SSFP sequences due to sclerosis [27, 28]. Thus, DWI is not of definite value in distinguishing these two entities.

### Chemical shift imaging

CSI was used to quantitatively assess vertebral bone marrow's fat and water content on a voxel-by-voxel-basis, done in two patients in our cohort [12]. In a previous study by Zajick et al. on 221 marrow lesions in 92 patients, a signal drop of more than 20% on out-of-phase images compared with in-phase images indicated benign lesions [13]. By benign lesions, all hematopoietic islands in our patient cohort showed signal drops greater than this 20% threshold on out-of-phase images due to their fat content. In contrast, normal fat-containing marrow is replaced with high cellular tumorous tissue in malignant lesions. This increase in water protons is associated with a lack of suppression on the out-of-phase images [13, 29, 30]. Thus, chemical shift imaging seems to be of value for the differentiation of hematopoietic islands of the axial skeleton and osteoblastic metastases.

### CT imaging

On CT, none of the lesions showed sclerosis, which contrasts with osteoblastic metastases [31]. Therefore, this is a powerful and essential imaging feature to make the correct diagnosis.

### Hybrid imaging techniques

^18^F-FDG PET/CT was done on two patients. No increased FDG- uptake was found in both patients (cut-off value SUV_max_ > 3). This is in contrast to a previous case report by Bordalo-Rodrigues et al. They reported a patient with lung carcinoma who received ^18^F-FDG PET/CT examination as pretherapeutic staging, where increased uptake was noted in a biopsy-proven hematopoietic island in the vertebral body of Th8 [9]. Considering this finding, it has to be stated that focal areas of normal but hypercellular red marrow may show increased uptake on FDG PET/CT and, therefore, may be confused with neoplasm or infection. This may be due to the upregulation of glucose transporters and metabolism in stimulated cells, also known for increased FDG-uptake in patients undergoing granulocyte-colony-stimulating-factor, which stimulates growth and differentiation of hematopoietic stem cells. However, in these cases- in contrast to hematopoietic islands- FDG-uptake is more diffuse [7, 8]. The hematopoietic activity in our lesions might have been too low to cause an increased FDG-uptake. In conclusion, it should be emphasized that hematopoietic islands may remain occult on FDG PET/CT. Therefore, this imaging modality is not of direct value for differentiating osteoblastic metastases and focal hematopoietic islands of the spine.

### Skeletal scintigraphy

Tc-99 m skeletal scintigraphy was performed in one patient in our cohort and was unremarkable. This contrasts with a previous report by Lee et al., who described an increased radiotracer accumulation in focal hematopoietic hyperplasia of the third right rib in a 24-year-old patient [11]. Thus, skeletal scintigraphy is not of value in distinguishing hematopoietic islands from osteoblastic metastases.

### Bone marrow biopsy

Five patients received CT-guided biopsy to confirm the diagnosis. All samples showed proportions of bone marrow with moderately increased hematopoiesis. The fat cell content was mildly reduced. There was no evidence of malignancy (Figs. 1f and 2g).

### Limitations

One limitation of our study is the small number of patients. However, so far, there is little literature available on this topic. In addition, diagnosis-confirming CT-guided biopsy was obtained in only five patients. However, osteoblastic metastases could be ruled out by follow-up MRI in the remaining cases. As another limitation, patients with osteoblastic metastases were not included in this study. Regarding typical image characteristics of osteoblastic metastases, a reference was made to previous work. Furthermore, imaging parameters varied slightly between patients, and not all patients received DWI, CSI, and hybrid imaging. However, standard morphological MRI sequences were available in all patients, and gadolinium was applied in most cases (nine out of ten patients).

## Conclusion

Hematopoietic islands of the axial skeleton are rare and may easily be confused with osteoblastic metastases due to similar signaling behavior on standard morphological MRI. We recommend performing MRI in combination with CT at the initial diagnosis since osteoblastic metastases are characterized by sclerosis, nor are hematopoietic islands, which typically do not show any bony texture changes. Another vital hint to making the correct diagnosis of hematopoietic islands versus osteoblastic metastases is the only moderate signal reduction on unenhanced T1-weighted images, typically seen in this entity. In addition, CSI seems to be of value in demonstrating the fat content in focal hematopoietic islands in contrast to osteoblastic metastases. Nevertheless, osteoblastic metastases should be conscientiously ruled out in particular when underlying malignancy is known. Therefore, in tumor patients respectively unclear cases, early histologic confirmation of the bone marrow lesion is reasonable, e.g., by CT-guided biopsy.

## Data Availability

The datasets analyzed during the current study are available from the corresponding author on reasonable request.

## Abbreviations

^18^F-FDG-PET/CT: ^18^F-fluorodeoxyglucose Positron Emission Tomography/Computed Tomography
Tc-99 m: Technetium-99 m
DWI: Diffusion-weighted imaging
MRI: Magnetic resonance imaging
SSFP: Steady-state free precession
STIR: Short tau inversion recovery
TSE: Turbo spin-echo
CSI: Chemical shift imaging
